# Local and systemic neutrophilic inflammation in patients with lung cancer and chronic obstructive pulmonary disease

**DOI:** 10.1186/1471-2172-14-36

**Published:** 2013-08-06

**Authors:** Neringa Vaguliene, Marius Zemaitis, Simona Lavinskiene, Skaidrius Miliauskas, Raimundas Sakalauskas

**Affiliations:** 1 Department of Pulmonology and Immunology, Medical Academy, Hospital of Lithuanian University of Health Sciences, Eiveniu 2, Kaunas LT-50028, Lithuania

**Keywords:** Lung cancer, Chronic obstructive pulmonary disease, Neutrophils, Reactive oxygen species

## Abstract

**Background:**

Recent investigations suggest that neutrophils play an important role in the immune response to lung cancer as well as chronic obstructive pulmonary disease (COPD). The aim of this study was to evaluate the amount of neutrophils and markers of their activity in lung cancer and COPD and in coexistence of these two diseases.

**Methods:**

In total, 267 persons were included in the study: 139 patients with lung cancer, 55 patients with lung cancer and COPD, 40 patients with COPD, and 33 healthy subjects. Peripheral blood and BAL fluid samples were obtained for cell count analysis and determination of NE, MPO levels and ROS production. NE and MPO levels in the serum and BAL fluid were determined by ELISA. ROS production was analyzed by flow cytometer.

**Results:**

The percentage, cell count of neutrophils and neutrophil to lymphocyte ratio in the peripheral blood were significantly higher in lung cancer patients with or without COPD compared to COPD patients or healthy individuals (P < 0.05). The percentage and cell count of neutrophils in BAL fluid were significantly lower in patients with lung cancer with or without COPD than in patients with COPD (P < 0.05). However, BAL fluid and serum levels of both NE and MPO were significantly higher in patients with lung cancer than COPD patients or healthy individuals (P < 0.05). Neutrophils produced higher amounts of ROS in patients with lung cancer with or without COPD compared with COPD patients or healthy individuals (P < 0.05).

**Conclusions:**

The results from this study demonstrate higher degree of local and systemic neutrophilic inflammation in patients with lung cancer (with or without COPD) than in patients with COPD.

## Background

Cancer is a multifactorial disease which is determined by complex interactions between genetic variants and environmental factors [1]. Various non-infectious chronic inflammatory conditions have been consistently associated with the increased risk of cancer development. Examples would be large bowel inflammatory conditions, which create a risk of colorectal cancer, chronic pancreatitis, which may precede the development of pancreatic cancer, and chronic obstructive pulmonary disease (COPD), which increases the risk of lung cancer [2]. However, there are many unanswered questions about the role of chronic inflammation in cancer development [2]. COPD, as well as lung cancer, are disorders characterized by an abnormal local and systemic inflammatory response with smoking as a major environmental risk factor [3].

Chronic inflammation involves activation and recruitment of leucocytes, especially neutrophils. Neutrophils are key blood cells, which respond immediately to inflammatory stimulus and contain a wide range of toxic compounds for pathogen removal [3]. Furthermore, the release of huge amounts of reactive oxygen species (ROS) by neutrophils plays a key role in enhancing the inflammation through the activation of mitogen-activated protein kinases and redox-sensitive transcription factors such as nuclear factor kappa B and activator protein-1 [4]. A small amount of ROS is essential for cell protection, viability and apoptosis. However, high amounts of ROS may act as carcinogenic agents by inducing structural changes in DNA and by modulating stress gene expression [5]. An oxidative stress is known to be increased in the cells of lung cancer patients and COPD patients, especially smokers [6-8]. Activated neutrophils express serine proteases, including neutrophil elastase (NE), cathepsin G, and proteinase-3, matrix metalloproteinases (MMP)-8, MMP-9, and proteins, such as myeloperoxidase (MPO) and human neutrophil lipocalin, and release them from the granules into the extracellular space. These mediators play an important role in the inflammatory process [9,10]. Neutrophil elastase is a neutral protease that is able to degrade insoluble elastin. The imbalance between proteases and their inhibitors (alpha 1-antitrypsin) may cause emphysematous changes in the lung tissue and the development of COPD. Furthermore, NE has also been shown to cleave cell surface epidermal growth factor and transfor growth factor-alpha [11]. The role of NE in the development of lung cancer has been described in animal models and cell-line studies [12,13], but there is limited data from the investigations of lung cancer patients. Myeloperoxidase is an endogenous oxidant enzyme, which plays an important role in bacterial killing by neutrophils and is involved in COPD pathogenesis [14]. In addition, there is evidence of MPO role in pathogenesis of lung cancer [15]. Neutrophil elastase and MPO are mostly released from activated neutrophils and act locally in the airways and other pulmonary compartments. However, these inflammatory mediators can be also detected in serum and be considered as parameters of systemic inflammation [10,14].

Local inflammation, the main characteristic feature of COPD, is associated with an infiltration of airway by inflammatory cells and an increased expression of cytokines, chemokines, enzymes, growth factors and adhesion molecules [3]. Bronchoalveolar lavage (BAL) is a useful procedure to sample the cellular and humoral constituents of the lung microenvironment. Although cellular changes in BAL fluid have been widely studied in COPD patients [16], data about cellular composition in lung cancer patients with COPD are lacking. There are also not enough data comparing the cellular changes in lung cancer and COPD.

A direct role of chronic inflammation in the pathogenesis of lung cancer and its relation to the processes in COPD is still not fully understood. Therefore, the aim of our study was to evaluate the local and systemic chronic inflammation by investigating the amount of neutrophils and markers of their activity (ROS, NE, MPO) in peripheral blood and BAL fluid of patients with lung cancer, COPD and having both diseases simultaneously.

## Results

### Characteristics of subjects

The clinical characteristics of the study population are described in Table 1. There were no significant age, BMI differences between the groups. However, significantly more lung cancer patients with COPD were current smokers. Smoking intensity did not differ when compared with other groups. FEV_1_ and FEV_1_/FVC did not differ between COPD groups. There were significantly fewer females in the COPD group compared with lung cancer and healthy individual groups.

**Table 1:** Characteristics of subjects

**Variable**	**Lung cancer group**		**Control group**	
	**Without COPD n = 139**	**With COPD n = 55**	**COPD n = 40**	**Healthy individuals n = 33**
Gender^a^				
Male	103 (74.1)	50 (90.9)	36 (90.0)	24 (72.7)
Female	36 (25.9)	5 (9.1)	4 (10.0)	9 (27.3)
Age, mean ± SEM, years	63.1 ± 0.9	64.5 ± 1.2	65.4 ± 1.2	62.6 ± 0.9
BMI, mean ± SEM, kg/m^2^	27.3 ± 0.4	26.9 ± 0.5	28.4 ± 0.6	26.7 ± 0.6
Smoking history^aa^				
Never smoker	30 (21.6)	0	0	11 (33.3)
Former smoker	27 (19.4)	7 (12.7)	19 (47.5)	10 (30.3)
Current smoker	82 (59.0)	48 (87.3)	21 (52.5)	12 (36.4)
Smoking pack-years, median (range):				
Former smoker	28.0 (10–54)	30.0 (15–40)	33.0 (13–57)	22.0 (11–44)
Current smoker	38.0 (10–60)	40.0 (12–60)	39.5 (10–94)	32.0 (20–56)
FEV_1,_ mean ± SEM, % pred	91.2 ± 1.7*	56.7 ± 1.6^§^	55.7 ± 2.3^§^	106.6 ± 3.0
FEV_1_/FVC ratio, mean ± SEM, % pred	96.6 ± 0.7*	70.6 ± 1.1^§^	74.0 ± 1.8^§^	104.8 ± 0.4

*FEV*_1_ forced expiratory volume in one second, *FVC* forced vital capacity, *BMI* body mass index, *COPD* chronic obstructive pulmonary disease.

*P < 0.05, when compared to lung cancer with COPD, COPD, and healthy individuals groups.

^§^P < 0.05, when compared with healthy individuals.

^a^χ^2^-10.61, P < 0.05.

^aa^χ^2^-48.14, P < 0.001.

The clinical characteristics of the patients with lung cancer are described in Table 2.

**Table 2:** Characteristics of patients with lung cancer at the time of diagnosis

**Variable**	**Patients, No (%)**
Histologic type:	
Squamous cell carcinoma	42 (21.6)
Adenocarcinoma	86 (44.3)
Large cell carcinoma	33 (17.0)
NSCLC-NOS	16 (8.2)
SCLC	17 (8.9)
Stage of disease:	
Stage I	13 (6.7)
Stage II	9 (4.6)
Stage III	59 (30.4)
Stage IV	113 (58.3)
ECOG performance status:	
0–1	165 (85.1)
2–3	29 (14.9)

*NSCLC* non-small cell lung cancer, *SCLC* small cell lung cancer, *NSCLC-NOS* non-small cell lung cancer not otherwise specified, *ECOG* Eastern Cooperative Oncology Group.

### Cellular patterns of peripheral blood and BAL fluid

Table 3 shows the cellular patterns of peripheral blood and BAL fluid from all the investigated groups. The percentage and cell count of leucocytes, neutrophils and monocytes in the peripheral blood were significantly higher in patients with lung cancer with or without COPD than in patients with COPD or healthy individuals (P < 0.05) (Table 3). Furthermore, the percentage and cell count of leucocytes and neutrophils were significantly higher in patients with COPD than healthy individuals. The cell count of lymphocytes did not differ between groups (P > 0.05). The neutrophil to lymphocyte (N/L) ratio was significantly higher in lung cancer patients with or without COPD compared to patients with COPD or healthy individuals (Table 3). The N/L ratio (3.28 ± 0.14 vs. 4.47 ± 0.03, P < 0.05) and neutrophil cell count (5.61 ± 0.19 vs. 6.83 ± 0.52, P < 0.05) were significantly higher in the lung cancer patients with poor performance status 2–3, when compared to the patients with performance status 0–1 as well as. There were no significant differences in leucocyte, neutrophil, monocyte cell counts and N/L ratio according to gender, stage and histologic type of the disease (data not shown).

**Table 3:** Total and separate cell percentage and counts in peripherial blood and BAL fluid

**Variable**	**Lung cancer group**		**Control group**	
	**Without COPD**	**With COPD**	**COPD**	**Healthy individuals**
Peripheral blood cells, mean ± SEM:				
Leukocytes, x10^9^l	8.57 ± 0.25*^§^	8.71 ± 0.48*^§^	5.81 ± 0.13*	5.35 ± 0.17
Neutrophils, %	65.34 ± 0.85*^§^	67.45 ± 1.43*^§^	61.77 ± 1.18*	56.92 ± 1.65
Neutrophils, x10^9^l	5.72 ± 0.22*^§^	5.96 ± 0.39*^§^	4.35 ± 0.22*	3.70 ± 0.25
Lymphocytes, %	23.01 ± 0.79*^§^	21.11 ± 1.21*^§^	25.93 ± 0.99*	31.64 ± 1.45
Lymphocytes, x10^9^l	1.88 ± 0.06	1.84 ± 0.10	1.78 ± 0.09	1.99 ± 0.09
Monocytes, %	8.60 ± 0.30*^§^	8.10 ± 0.49*^§^	6.82 ± 0.29	6.26 ± 0.42
Monocytes, x10^9^l	0.87 ± 0.09*^§^	0.88 ± 0.17*^§^	0.46 ± 0.03	0.40 ± 0.03
Neutrophil/lymphocyte ratio, median (range)	2.92(0.93–13.42)*^§^	3.08(1.18–8.84)* ^§^	2.35(1.13–4.25)^§^	1.86(1.16–3.21)
BAL fluid cells, mean ± SEM:				
Neutrophils, %	8.01 ± 1.37*^§^	7.94 ± 0.83*^§^	14.10 ± 2.96*	1.29 ± 0.21
Neutrophils, x10^6^/ml	0.39 ± 0.12*^§^	0.41 ± 0.15*^§^	0.54 ± 0.22*	0.08 ± 0.05
Macrophages, %	73.59 ± 1.51^§^	72.89 ± 1.71^§^	65.50 ± 3.19*	74.40 ± 1.77
Macrophages, x10^6^/ml	3.24 ± 0.20^§^	3.15 ± 0.30^§^	2.95 ± 0.65*	3.21 ± 0.51
Lymphocytes, %	18.21 ± 1.02	18.81 ± 1.48	19.99 ± 1.80	21.97 ± 1.62
Lymphocytes, x10^6^/ml	0.87 ± 0.34	1.03 ± 0.37	1.11 ± 0.53	0.63 ± 0.26
Eosinophils, %	0.29 ± 0.04	0.36 ± 0.06	0.41 ± 0.15	0.34 ± 0.11
Eosinophils, x10^6^/ml	0.02 ± 0.01	0.01 ± 0.01	0.03 ± 0.01	0.02 ± 0.01

Data are expressed as mean ± SEM. *P < 0.05 when compared with healthy individuals; ^§^P < 0.05 when compared to COPD group.

The percentage and cell count of neutrophils were significantly higher in the patients with lung cancer with or without COPD than in healthy individuals but significantly lower than in patients with COPD (P < 0.05). The percentage and cell count of macrophages was significantly higher in lung cancer groups and healthy individuals, than in COPD group (P < 0.05).

### Neutrophil ROS production *in vitro*

**Figure 1:**
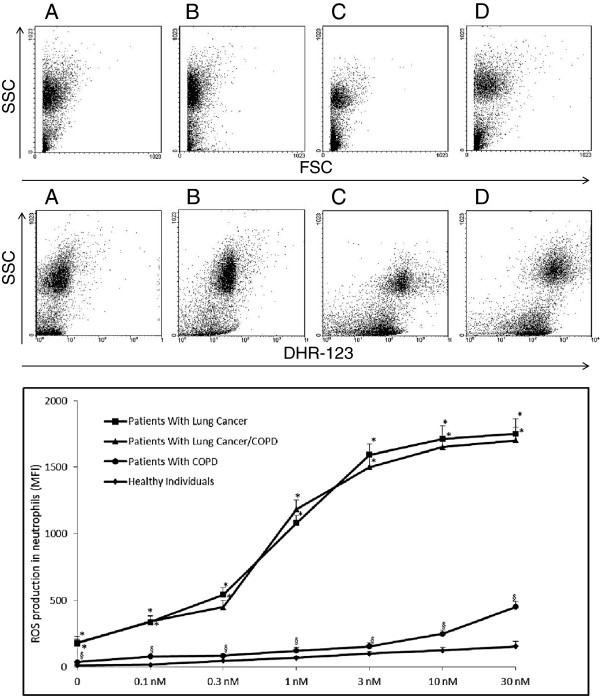
**A histogram representing changes in ROS production in the neutrophils of peripheral blood of study patients after stimulation with different concentrations of PMA.** Data are presented as mean fluorescence intensity (MFI) ± SEM. *P < 0.05, when compared with COPD and healthy individuals groups; ^§^P < 0.05, when compared with healthy individuals. The representative dot plot of neurophil population isolated from peripheral blood of healthy individuals **(A)**, patients with COPD **(B)**, patients with lung cancer/COPD **(C)** and patients with lung cancer **(D)** stimulated upon 30 nM of Phorbol 12-myristate 13-acetate (PMA). Side light scater (SSC) represents the granularity, complexity of the cells; FSC – forward light scatter (FSC) represents cell size; dihydrorhodamine-123 (DHR-123) a green fluorescent compound showing H_2_O_2_ intensity in neutrophils.

Neutrophils produced higher spontaneous ROS levels in the groups of lung cancer patients with or without COPD compared to the COPD patients or healthy individuals (P < 0.05) (Figure 1). The spontaneous ROS production in the lung cancer patients did not significantly differ despite the coexistence of COPD. Spontaneous ROS production in neutrophils did not differ between the male and female patients with lung cancer (175.53 ± 2.12 MFI vs. 175.29 ± 3.78 MFI, P > 0.05). There were no significant differences of spontaneous ROS production in neutrophils among the major histologic types of lung cancer: squamous cell carcinoma, adenocarcinoma, large cell carcinoma, non-small cell lung cancer not otherwise specified and small cell carcinoma (166.53 ± 4.93 MFI, 176.50 ± 2.73 MFI, 172.29 ± 4.56 MFI, 182.47 ± 6.43 MFI, 180.53 ± 3.98 MFI, P > 0.05) as well as between non-small cell lung cancer and small cell lung cancer groups (174.67 ± 2.02 MFI vs. 180.53 ± 3.98 MFI, P > 0.05). Additionally, spontaneous ROS production in neutrophils was significantly higher in patients with advanced lung cancer than in those with early lung cancer (183.66 ± 1.78 MFI vs. 145.91 ± 2.67 MFI, P < 0.001) and in the lung cancer patients with poor performance status 2–3 compared with those with performance status 0–1 (209.10 ± 4.93 MFI vs. 169.37 ± 1.52 MFI, P < 0.001). Furthermore, the patients with early lung cancer had a significantly higher spontaneous ROS production in neutrophils than the patients with COPD (P < 0.01). There were no significant differences of spontaneous ROS production in neutrophils in lung cancer groups among never smokers, former and current smokers (174.75 ± 4.93 MFI, 181.00 ± 5.65 MFI, 174.33 ± 2.19 MFI, P > 0.05). Additionally, spontaneous ROS production was found to be higher in lung cancer patients with or without COPD, who have never smoked, when comparing to current smokers with COPD (172.86 ± 5.26 and 179.25 ± 3.68 vs. 64.29 ± 1.17, P < 0.001). Different concentrations of PMA (0.3-30 nM) stimulated ROS production in neutrophils in all studied groups (Figure 1). An obvious increase of ROS production in neutrophils was detected after stimulation with 0,3 nM of PMA in lung cancer patients (with and without COPD), and with 10 nM in COPD group (P < 0.05). But the most significant increase of ROS production in all studied groups was observed in neutrophils stimulated with 30 nM PMA. There were no correlations between spontaneous ROS production in neutrophils and age, BMI and smoking intensity in study groups (data not shown).

### Levels of NE and MPO

**Figure 2:**
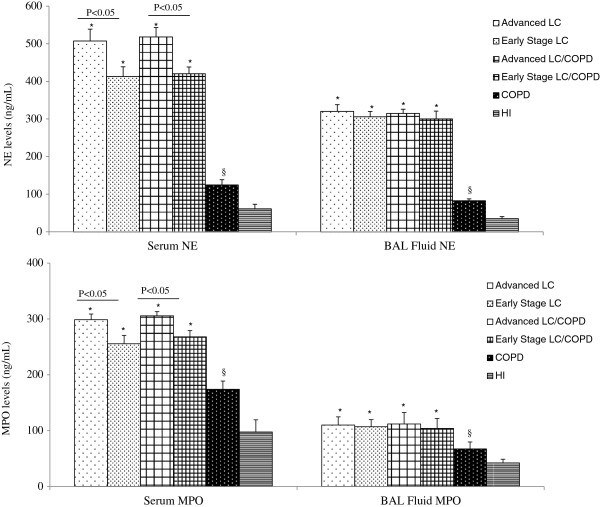
**NE and MPO levels (ng/mL) in serum and BAL fluid in patients with advanced and early stage lung cancer, patients with lung cancer/COPD, patients with COPD and healthy individuals.** Data are presented as mean ± SEM. *P < 0.05, when compared with COPD and healthy individuals; ^§^P < 0.05, when compared with healthy individuals.

Serum and BAL fluid levels of both NE and MPO were significantly higher in patients with lung cancer than in patients with COPD or healthy individuals (P < 0.05) (Figure 2). However, serum and BAL fluid NE and MPO levels did not significantly differ in lung cancer groups (with and without COPD) (P > 0.05). We did not find any significant differences of NE and MPO levels in serum and BAL between the male and female patients with lung cancer (NE serum 518.64 ± 10.38 ng/mL vs. 489.22 ± 15.17 ng/mL, NE BAL fluid 297.34 ± 9.88 ng/mL vs. 327.77 ± 18.79 ng/mL, MPO serum 297.56 ± 3.22 ng/mL vs. 301.54 ± 15.80 ng/mL, MPO BAL fluid 111.69 ± 13.64 ng/mL vs. 112.83 ± 10.76 ng/mL, P > 0.05). There were no significant differences of NE and MPO levels in serum and BAL fluid between the non-small cell lung cancer and small cell lung cancer (NE serum 512.19 ± 8.99 ng/mL vs. 537.72 ± 11.97 ng/mL, NE BAL fluid 316.53 ± 8.43 ng/mL vs. 332.65 ± 25.03 ng/mL, MPO serum 298.35 ± 13.16 ng/mL vs. 305.31 ± 17.32 ng/mL, MPO BAL fluid 114.09 ± 14.08 ng/mL vs. 130.83 ± 10.91 ng/mL, P > 0.05) as well as among various histological types of cancer (data not shown). However, serum NE and MPO levels were significantly higher in patients with advanced lung cancer than in those with early lung cancer (P < 0.05) (Figure 2). Furthermore, patients with early lung cancer had a significantly higher NE levels than patients with COPD (P < 0.01).There were no significant differences of NE and MPO levels in serum and BAL fluid in lung cancer patients with performance status 0–1 when comparing to patients with performance status 2–3 (NE serum 509.51 ± 8.76 ng/mL vs. 528.01 ± 18.45 ng/mL, NE BAL fluid 314.38 ± 8.29 ng/mL vs. 338.21 ± 25.08 ng/mL, MPO serum 297.13 ± 13.00 ng/mL vs. 300.85 ± 18.03 ng/mL, MPO BAL fluid 112.94 ± 14.14 ng/mL vs. 121.72 ± 11.92 ng/mL, P > 0.05). There were no differences of NE and MPO levels in lung cancer groups (with or without COPD) among subjects that have never smoked, former and current smokers (P > 0.05). Additionally, NE and MPO levels in serum and BAL fluid were found to be significantly higher in the lung cancer patients, who have never smoked compared with the current smokers with COPD (NE serum 480.11 ± 19.05 vs. 132.51 ± 18.72, MPO serum 286.96 ± 9.94 vs. 183.42 ± 11.95, MPO BAL fluid 103.65 ± 5.19 vs. 68.43 ± 7.59, P < 0.05). Correlations between NE, MPO levels and spontaneous ROS production in the peripheral blood neutrophils in patients with lung cancer are presented in Table 4. No correlations were found between serum and BAL fluid NE, MPO levels and age, BMI, smoking intensity in the investigated groups (data not shown).

**Table 4:** Correlation between serum and BAL fluid NE, MPO levels and spontaneous ROS production in peripheral blood neutrophils in patients with lung cancer

	**Spontaneous ROS production**	
	**r**	**p**
NE serum	0.396	0.007
NE BAL fluid	0.461	0.004
MPO serum	0.428	0.005
MPO BAL fluid	0.382	0.007

*NE* neutrophil elastase, *MPO* myeloperoxidase, *BAL fluid* bronchoalveolar lavage fluid.

## Discussion

The goal of this study was to investigate local and systemic neutrophilic inflammation in patients with lung cancer and COPD considering the common inflammatory signaling pathway in both diseases. It is known that chronic inflammation plays a role in pathogenesis of both diseases there are limited integrated data comparing chronic inflammatory processes when there is coexistence of lung cancer and COPD. It is known that COPD is a major independent risk factor for lung cancer among smokers and about 50-90% of patients with lung cancer also have COPD [17]. A mechanism, explaining why smokers with COPD have an increased risk for lung cancer when compared to smokers without COPD, is still not clear. Furthermore, it is still not known why some patients with COPD get lung cancer and some patients don't. In order to understand the inflammatory mechanisms and associations between lung cancer and COPD we aimed to analyze the patterns of local (BAL fluid) and systemic (peripheral blood) chronic neutrophilic inflammation in lung cancer and COPD.

Our results are consistent with the findings of previous reviews, showing that the count of neutrophils in peripherial blood was higher in lung cancer patients [18] as well as in patients with COPD [19] when compared to healthy individuals. To our knowledge such data comparing neutrophil cell count and N/L ratio in lung cancer patients, patients with COPD and both diseases in coexistence are presented for the first time. Recent studies have shown that the N/L ratio has a significant prognostic value for chronic conditions such as hypertension, diabetes mellitus [20], many cancers as well as lung cancer [21-24]. We demonstrated an increased neutrophil cells count and N/L ratio in peripheral blood in patients with lung cancer compared with COPD patients, but did not find significant difference between the groups of lung cancer patients with COPD and without COPD. Furthermore, N/L ratio was significantly higher in patients with COPD than in healthy subjects. The development of chronic systemic neutrophilia in cancer, as well as in COPD, has not been fully understood. There are some hypotheses explaining pathogenetic mechanisms, which are responsible for the increased neutrophil count in the peripheral blood. One of them, states that increased neutrophil count in peripheral blood in case of COPD can be influenced by the airway inflammation. This enhanced neutrophil influx to the airways may stimulate the activation of inflammatory markers, which on the other hand activate the neutrophils, cause an increase in their vitality and migration to the lung tissue due to chemoattractants [25]. The other hypothesis, suggests that an enhanced expression of granulocyte-macrophage colony-stimulating factor and IL-6 in patients with COPD [19,26] plays and important role, as these mediators stimulate neutrophil release from the bone marrow, causing the increased neutrophil count in the peripheral blood. The same mechanisms can be responsible for the increased numbers of neutrophils and N/L ratio in lung cancer as well. However, there is additional mechanism that could contribute to the increase in neutrophil count. Cancer cells produce granulocyte colony-stimulating factor, which directly stimulates bone marrow to release neutrophils [27]. Because of these mechanisms, lung cancer patients have higher neutrophil count and more pronounced systemic chronic inflammation than patients with COPD.

There are limited data comparing local inflammation in lung cancer patients and COPD. Analysis of BAL fluid cells and extracellular components allows to describe immune processes in the airway microenvironment. In agreement with the data from other studies, our results revealed that percentage and cell count of neutrophils were significantly higher in lung cancer patients [28,29] and COPD patients [16] compared with healthy subjects. In contrast to neutrophilia in peripheral blood, percentage of neutrophils in BAL fluid was significantly lower and percentage of macrophages was significantly higher in lung cancer patients compared with COPD patients. Furthermore, we observed that in lung cancer patients, when compared with COPD patients, not only the count of macrophages in BAL fluid was increased, percentage and cell count of monocytes in peripheral blood were also higher. Lung cancer cells generate chemotactic factors and chemokine ligand for circulating monocytes, which are recruited into the lungs, where they differentiate into the macrophages [30]. It is known that immune cells, including macrophages, infiltrate the tumor stroma [2,31]. It is well know that monocyte-lineage cells including macrophages also play an important role in the pathogenesis of COPD [3]. A plausible explanation for the increased count of macrophages in BAL fluid of patients with lung cancer is that tumor promotes migration of monocytes and their differentiation into the macrophages. This confirms the fact, that macrophages play central role in the immune response to lung cancer.

We observed that mean serum and BAL fluid levels of NE and MPO were significantly higher in patients with lung cancer (with or without COPD) compared with COPD patients and healthy subjects. Neutrophil elastase and MPO are important markers of neutrophil activation and neutrophilic inflammation [9,10]. Some studies have identified increased NE and MPO levels in patients with COPD [14] and healthy smokers [10]. The role of NE and MPO in chronic lung inflammation and association with lung cancer development has been characterized in cell line and animal models [11-13,32]. Nevertheless, there are no clear data comparing the importance of NE and MPO in lung cancer and COPD. Although we demonstrated that neutrophil cell count and percentage in BAL fluid were lower in lung cancer groups compared with COPD patients, NE and MPO levels in BAL fluid were significantly higher in lung cancer groups than in COPD group. Thus, our findings let us hypothesize that in patients with lung cancer neutrophils appear in a more activated state through the production of NE, MPO than in patients with COPD. Furthermore, there are some data showing that NE and MPO is expressed not only in neutrophils but also in monocyte-derived macrophages [33].

It is generally accepted that oxidative stress, which is promoted by cigarette smoke, may be involved in cancer development [2,5]. The literature is full of data on the increased oxidative stress in patients with COPD [3,7]. However it is still little known about ROS production in lung cancer patients with COPD and data comparing lung cancer with COPD are lacking. In the present study we demonstrated that spontaneous ROS production in peripheral blood neutrophils was higher in both lung cancer groups (lung cancer and lung cancer with COPD) than in patients with COPD, but there were no differences between lung cancer patients with COPD or without COPD. Heijink and colleagues suggested that chemical factor like cigarette smoke may influence a more intensive ROS production in COPD and healthy individuals [34]. As a chemical factor which causes neutrophil activation we used a PMA. Our study showed that PMA induced intensive ROS production not only in patients with COPD and healthy individuals but in lung cancer patient groups (with and without COPD) as well. Our results revealed that activated neutrophils after stimulation with PMA produced more ROS in lung cancer patients (with and without COPD) compared with COPD patients. It means that chemical factors cause enhanced inflammation in lung cancer. In addition, we provided evidence that intracellular ROS was increased in peripheral blood neutrophils of lung cancer patients which positively correlated with levels of both inflammatory markers (NE, MPO). Such correlation supports the hypothesis that intensive chronic neutrophilic inflammation promotes more intensive ROS production in lung cancer compared to COPD.

We also investigated levels of NE, MPO and ROS production in the patients with different stage of lung cancer (early vs. advanced). Our data demonstrated that patients with advanced lung cancer had significantly higher serum NE, MPO levels and more intensive ROS production in peripheral neutrophils than patients with early stage lung cancer. These results are consistent with the results reported in other studies [11], presenting additional evidence of the importance of chronic neutrophilic inflammation in lung cancer progression [2]. Furthermore, our findings of higher systemical NE, MPO and ROS production in patients with early lung cancer compared with COPD patients further support the fact, that chronic inflammation can be more pronounced in lung cancer than COPD.

Additional studies have demonstrated that smoking stimulates not only local but also systemic inflammation [6,16]. However, the data on the influence of smoking itself on chronic neutrophilic inflammation in lung cancer patients with coexisting COPD are scarce. The data are contradictory, some researches have shown that cigarette smoke is strongly associated with the increased inflammation of airways, proved by the exhaled breath condensate in lung cancer patients [35]. Other study failed to prove these associations in lung cancer patients [36]. There is evidence that cigarette smoking plays an important role as the starting point of chronic inflammation [6] but has little influence in promoting inflammation in lung cancer. However, our results indicate that in patients with lung cancer local and systemic inflammation was increased independent of smoking and not only cigarette smoking but also other factors play a significant role in promotion of chronic inflammation in lung cancer.

## Conclusions

We observed that individuals with lung cancer (with or without COPD) had more pronounced local and systemic neutrophilic inflammation in comparison with patients with COPD and healthy subjects. These data permit a suggestion that neutrophilic chronic inflammation has a significant role in lung cancer pathogenesis and is more pronounced in patients with lung cancer than patients with COPD.

## Methods

### Patients

We investigated 267 subjects: 139 patients with lung cancer, 55 lung cancer patients with stable moderate to severe COPD, 40 patients with stable moderate to severe COPD and 33 healthy individuals at the Hospital of Lithuanian University of Health Sciences Kaunas Clinics from 2009 April to 2012 May. All patients met following criteria: did not use inhaled or systemic steroids at least 1 month before the study and had no clinical or radiological evidence of infection. Subjects were excluded if they had a history of another malignancy or other diseases associated with systemic inflammation, such as rheumatoid arthritis, inflammatory bowel disease or connective tissue disorders. All lung cancer patients had histologically confirmed disease according to the WHO classification. The clinical stage, tumor type, and performance status (according to Eastern Cooperative Oncology Group (ECOG)) [37] of patients with lung cancer were recorded at the time of diagnosis, before administering anti-cancer therapy. Lung cancer stage was determined according to the *TNM Classification of Malignant Tumors,* the Seventh Edition [38]. Patients with lung cancer were divided into two groups according to the stage of disease: early lung cancer – patients with stage I-II cancer, and advanced lung cancer – patients with stage III-IV cancer. COPD was diagnosed according to the criteria of the Global Initiative for Chronic Obstructive Lung Disease (GOLD) [39]. At the time of evaluation, COPD patients were clinically stable (no exacerbations during the previous 2 months). All COPD patients were screened for deficiency of alpha-1 antitrypsin (AAT) by quantitative ELISA test (Eurodiagnosta, Sweden). Peripheral venous blood samples and bronchoscopy with BAL processing were obtained before administering anti-cancer therapy.

Study subjects according to their smoking status were divided into 3 categories: current smokers – smoking persons, having more than 10 pack-years smoking history; former smokers – individuals, having more than 10 pack-years smoking history, who had ceased smoking more than 2 years before the study; and never smokers – individuals who have never smoked [16]. Smoking history was calculated in pack-years as the product of tobacco use (in years) and the average number of cigarettes smoked per day/20 (years × cig. per day/20).

Body mass index (BMI) was calculated as measured weight (kg) divided by the square of measured height (m)^2^.

Kaunas Regional Ethics Committee for Biomedical Research approved the study and written informed consent was received from all participants.

### Lung function testing

Lung function was tested using a pneumotachometric spirometer “CustovitM” (Custo Med, Germany) with subjects in the sitting position. The highest value of forced expiratory volume in 1 second (FEV_1_) and forced vital capacity (FVC) from at least two technically satisfactory maneuvers differing by less than 5% were recorded. Predicted values were obtained from Quanjer et al. [40]. The subjects had to avoid the use of short-acting β_2_-agonists at least 8 h prior the test.

### BAL processing

BAL was performed in 76 patients with lung cancer, 43 patients with lung cancer and COPD, 37 patients with COPD and 28 healthy individuals, using fiberoptic bronchoscopy according to ERS guidelines for measurement of a cellular components and recommendations for standardization of BAL [41]. No serious complications occurred during or after the bronchoscopies. Before BAL procedure, the local upper airways anesthesia with 5 mL of 2% lidocaine (Grindex, Latvia) was performed. All bronchoscopy examinations were scheduled in the morning. In the group of patients with lung cancer BAL samples were taken during the diagnostic procedure from the lobe affected by the tumor (in cases with peripheral mass) or from the lobe adjacent to the affected lobe (in cases with a central mass) [28]. In patients with COPD and healthy individuals BAL samples were taken from the middle lobe [41]. The bronchoscope (Olympus, USA) was wedged into the segmental bronchus and 20 mL × 7, a total of 140 mL, of sterile saline solution (0.9% NaCl) was infused. Fluid was gently aspirated immediately after the infusion and was collected into a sterile container. The fluid was immediately filtered using 40 μm cell stainer (Becton Dickinson, USA) and centrifuged at 4°C for 10 min. Supernatants were removed and frozen at −70°C for further ELISA analysis. BAL cytospins were prepared using a cytocentrifuge (Shandon Southern Instruments, USA).

### BAL cell analysis

Prepared cytospins from the BAL fluid were stained using the May-Grünwald/Giemsa method for differential cell counts. Then 400 nonsquamous cells were counted per slide. The type of cell was identified using standard morphological criteria. Percentage (percentage of total nonsquamous cells) and absolute values (10^6^/mL) of all cell counts were recorded.

### Serum processing

Peripherial blood from all tested patients was collected into sterile tubes without additives (2×5 mL) and stored at room temperature for the surface clot formation (about 30 min). Then tubes were centrifuged at 1000 × g for 10 min at room temperature. From the upper layer of the sample the serum was vacuumed into sterile cold-resistant Eppendorf tubes and stored at −70°C for further ELISA analysis.

### Detection of serum and BAL fluid NE and MPO

NE and MPO levels in serum and BAL fluid were measured by enzyme-linked immunosorbent assay (ELISA) according to the manufacturer’s instructions (IBL International, USA and Immundiagnostik AG, Germany, respectively). The minimum detectable dose was 0.16 ng/mL for NE and 0.014 ng/mL for MPO. The concentration of NE and MPO in the samples was determined by comparing the optical density values of the samples to the standard curve.

The peripheral blood cell analysis was performed on an automated haematology analyser (Sysmex XE-5000, Japan). Neutrophil to lymphocyte ratio was calculated as the absolute neutrophil count divided by absolute lymphocyte count.

### Peripheral blood collection and isolation of neutrophils

Peripheral blood samples for neutrophil isolation were collected into sterile vacutainers with ethylene diamine tetraacetic acid (EDTA). Neutrophils were isolated by high density gradient centrifugation. The whole blood was layered on Ficoll-Paque PLUS (GE Healthcare, Finland) and centrifuged at 1000 g for 30 min at room temperature. Neutrophil population was separated by hypotonic lysis of erythrocytes. Isolated neutrophils were diluted in cell culture RPMI 1640 media (Biological Industries, Israel), at a final concentration of 2 × 10^6^/ml. The viability of neutrophils was checked by flow cytometry and it always was > 95%.

### *In vitro* ROS production in neutrophils

ROS production in neutrophils was induced by chemical (phorbol 12-myristate 13-acetate (PMA), 0.1, 0.3, 1, 3, 10, 30 nM) factor in sterile 96-well microplates (Falcon). For the detection of generated ROS, a nonfluorescent dye, dihydrorhodamine-123 (DHR-123, final concentration, 750 ng/mL; Invitrogen, USA), was added. DHR-123 interacts with intracellular ROS and is oxidized to green-fluorescent fhodamine 123 (catalysed by cellular myeloperoxidase, MPO). Plates filled with different concentrations of PMA and neutrophil suspension were incubated for 45 min. at 37°C, 5% CO2. The relative amount of generated ROS was measured by flow cytometry determining the mean fluorescence intensity in neutrophil population (excitation wavelength, 488 nm).

### Flow cytometric analysis

Flow cytometric measurements were performed with a FACSCalibur cytometer (Becton Dickinson, USA). For each determination at least 10^4^ events were acquired. Characteristic (size and granularity/complexity) properties of neutrophils served to determine the purity of cellular suspensions and to monitor morphological changes after incubation and stimulation with tested substances. The total number of viable neutrophils was quantified and identified by adding propidium iodide (2 mg/mL, Calbiochem, Germany), which stains deoxyribonucleic acid of dead cells. Data were analyzed with the software CellQuest.

### Statistical data analysis

Statistical analysis of data was performed using the statistical SPSS for Windows 20.0 software package. Data are presented as means ± standard error of the mean and median with data range. The categorical data were analyzed using of the chi-square (χ^2^) test. For comparisons of data in more than two groups we used ANOVA or Kruskal-Wallis tests and in two groups - the Student *t* test or Mann–Whitney test for parametrically or nonparametrically distributed data respectively. The association between the levels of inflammation markers and clinicopathological characteristics was measured by the Pearson correlation coefficient.

## Abbreviations

BAL: Bronchoalveolar lavage; BMI: Body mass index; COPD: Chronic obstructive pulmonary disease; ECOG: Eastern Cooperative Oncology Group; FEV1: Forced expiratory volume in 1 sec; FVC: Forced vital capacity; MPO: Myeloperoxidase; NE: Neutrophil elastase; PMA: Phorbol 12-myristate 13-acetate; ROS: Reactive oxygen species.

## Competing interests

The authors declare that they have no competing interests.

## Authors’ contributions

MZ conceived the study design and coordinated the work. NV carried out the collection of data and drafted the manuscript. NV and SL performed the laboratory experiments. NV and MZ contributed to the analysis and interpretation of the data. SM and RS participated in the study coordination and revised the manuscript. All authors read and approved the final manuscript.
